# COVID-19 Pulmonary Pathology: The Experience of European Pulmonary Pathologists throughout the First Two Waves of the Pandemic

**DOI:** 10.3390/diagnostics12010095

**Published:** 2022-01-01

**Authors:** Francesco Fortarezza, Federica Pezzuto, Paul Hofman, Izidor Kern, Angel Panizo, Jan von der Thüsen, Sergei Timofeev, Gregor Gorkiewicz, Sabina Berezowska, Laurence de Leval, Cristian Ortiz-Villalón, Francesca Lunardi, Fiorella Calabrese

**Affiliations:** 1 Department of Cardiac, Thoracic, Vascular Sciences and Public Health, University of Padova Medical School, Via A. Gabelli 61, 35121 Padova, Italy; francesco.fortarezza@unipd.it (F.F.); federica.pezzuto@unipd.it (F.P.); francesca.lunardi@unipd.it (F.L.); 2 Laboratory of Clinical and Experimental Pathology, FHU OncoAge, Biobank BB-0033-00025, University Côte d’Azur, 06100 Nice, France; hofman.p@chu-nice.fr; 3 Department of Pathology, University Clinic of Respiratory and Allergic Diseases, 4204 Golnik, Slovenia; izidor.kern@klinika-golnik.si; 4 Complejo Hospitalario de Navarra, 31008 Pamplona, Navarra, Spain; angel.panizo@gmail.com; 5 Department of Pathology, Erasmus MC, 3015 Rotterdam, The Netherlands; j.vonderthusen@erasmusmc.nl; 6 Moscow City Hospital 40, 81146 Moscow, Russia; serg.timofeev@icloud.com; 7 Institute of Pathology, Medical University of Graz, 8036 Graz, Austria; gregor.gorkiewicz@medunigraz.at; 8 Institute of Pathology, Lausanne University Hospital and University of Lausanne, Rue du Bugnon 25, 1011 Lausanne, Switzerland; sabina.berezowska@chuv.ch (S.B.); Laurence.DeLeval@chuv.ch (L.d.L.); 9 Department of Pathology, Karolinska University Hospital, 17177 Solna, Sweden; cristian.ortiz-villalon@ki.se

**Keywords:** COVID-19, SARS-CoV-2, autopsy, lung pathology, CAPA

## Abstract

Autoptic studies of patients who died from COVID-19 constitute an important step forward in improving our knowledge in the pathophysiology of SARS-CoV-2 infection. Systematic analyses of lung tissue, the organ primarily targeted by the disease, were mostly performed during the first wave of the pandemic. Analyses of pathological lesions at different times offer a good opportunity to better understand the disease and how its evolution has been influenced mostly by new SARS-CoV-2 variants or the different therapeutic approaches. In this short report we summarize responses collected from a questionnaire survey that investigated important pathological data during the first two pandemic waves (spring-summer 2020; autumn-winter 2020–2021). The survey was submitted to expert lung pathologists from nine European countries involved in autoptic procedures in both pandemic waves. The frequency of each lung lesion was quite heterogeneous among the participants. However, a higher frequency of pulmonary superinfections, both bacterial and especially fungal, was observed in the second wave compared to the first. Obtaining a deeper knowledge of the pathological lesions at the basis of this complex and severe disease, which change over time, is crucial for correct patient management and treatment. Autoptic examination is a useful tool to achieve this goal.

## 1. Introduction

Coronavirus disease-19 (COVID-19), caused by the novel severe acute respiratory syndrome coronavirus 2 (SARS-CoV-2), has quickly spread after its outbreak in late 2019. There has been mainly a two-wave pattern of reported cases in several countries, with the first wave occurring during the period from spring to summer 2020 and the second in autumn and winter of the same year [1]. During the first wave the clinical phenotype of COVID-19 pneumonitis was reported as a heterogeneous disease from a mild-flu-like illness to severe pneumonitis with profound hypoxemia requiring mechanical ventilation. One of the striking features observed in severe COVID-19 was the coagulopathy with associated high incidence of thrombotic complications. Different clinical phenotypes have been reported: patients with features of classical acute respiratory distress syndrome (ARDS) and others with increased D-dimer concentrations [2]. Pathological studies mainly performed on autoptic samples described several lesions in different compartments (mainly alveolar and vascular) thus documenting a complex pathological scenario [3,4]. The analytic descriptions of lung lesions mainly detected at autopsy have progressively reinforced the awareness of the need for more specific treatments, such as the adaption of low-molecular weight heparin thrombophylaxis (for microthrombosis) in patients with increased D-dimer concentrations and the use of steroids (for a “cytokine storm”) in critically ventilated patients [5].

Probably due to the relaxation of prevention measures during the summer months, a second wave started in mid-September 2020 and extended until spring 2021. Comparing patients in first and second waves is difficult due to the logistical capacity of countries to detect and diagnose patients who were asymptomatic or had mild symptoms. However, most reports coming from hospitalized patients indicated a quite different clinical phenotype, often younger, with fewer comorbidities and with different clinical symptoms— renal and gastrointestinal disorders occurred more frequently than respiratory symptoms [6]. However, this clinical phenotype was not observed in all countries. For example, in Northern Italy no differences were observed in relation to gender, age, and number of comorbidities [7]. During the second wave, there was a remarkably frequent association of a fungal superinfection in patients with severe COVID-19 disease, named COVID-19-associated pulmonary aspergillosis (CAPA) [8]. CAPA was highly suspected and clinically diagnosed as probable and possible or was found post-mortem in some autoptic studies [9]. This is an important additional risk factor for a worse prognosis.

Studies focusing on the comparative evaluation of lesions are largely missing. This is especially true for autoptic comparative lung studies in patients who died from COVID-19 pneumonitis. The aim of the study was to evaluate the differences between COVID-19-related pathological lung lesions in the first and second waves through a survey sent to different European centers with considerable experience in pulmonary pathology involved in the COVID-19 autopsies.

## 2. Materials and Methods

**Figure 1:**
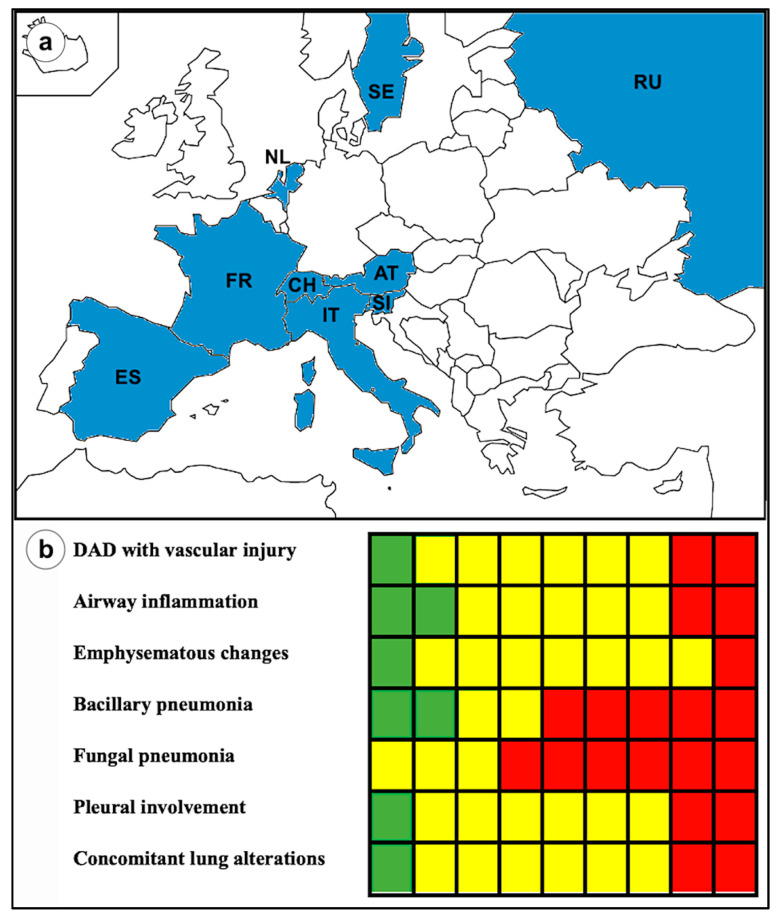
The European countries participating in the survey were Austria (AT), France (FR), Italy (IT), the Netherlands (NL), Russia (RU); Slovenia (SI), Spain (ES), Switzerland (CH), and Sweden (SE) (**a**). The frequency of pathological COVID-19-associated lung lesions during the two pandemic waves (**b**). Each box represents the answer of each participant. Green: more frequent during the first wave. Red: more frequent during the second wave. Yellow: equally present during both waves.

Data from COVID-19 autopsies were collected through a questionnaire survey. The electronic template was submitted via email to nine pathologists, members of the European Society of Pathology–Pulmonary Pathology Working Group, experts in thoracic pathology and involved in autoptic activities during the COVID-19 pandemic. The involved centers included: University Hospital of Padova (Italy); University Côte d’Azur (France); University Clinic of Respiratory and Allergic Diseases (Slovenia); Hospital of Navarra (Spain); Erasmus MC (The Netherlands); Moscow City Hospital #40 (Russia); Medical University of Graz (Austria); Lausanne University Hospital (Switzerland); Karolinska University Hospital (Sweden) (Figure 1a). For all centers, no specific inclusion criteria were used for patient enrollment in this study. Autopsies are usually performed to confirm dubious diagnoses and specific clinical-scientific questions. Particularly, the autopsies were performed for the patients who had shown an unusual clinical course. Participants were asked to provide data regarding their COVID-19 autoptic practice during the first (from March to August 2020) and the second wave (from September 2020 to February 2021) of the pandemic. Specifically, participants were asked to report the number of autopsies performed in these two different periods and provide: demographic data; technical procedures of autopsy and lung sampling; and the frequency of occurrence of several pathological signs related to COVID-19 lung-related pathological lesions (e.g., diffuse alveolar damage [DAD] with vascular impairment, airway inflammation, emphysematous changes with large bullae, bacillary and fungal pneumonia, concomitant lung lesions such as cancer or interstitial lung disease, and pleural involvement). The emphysematous changes analyzed in the study include those putatively related to severe COVID-19, characterized by the presence of large bullae, most likely due to the action of barotrauma (or other causes) on a pulmonary parenchyma made fragile by viral replication. The histological changes of bacillary pneumoniae include the patchy intra-alveolar and/or peri-bronchial fibrinopurulent exudate with predominant neutrophil granulocytes in addition to the presence of bacterial microorganisms better highlighted with special stains (Gram stain). Fungal pneumonia consists of an inflammatory and infectious pulmonary process, in which hyphae or fungal spores are histologically highlighted. In particular, the characteristics of *Aspergillus* hyphae are of an acute angle (45°) or dichotomous branching, septation, with a thickness of 2.5–4.5 µm. The microorganism was better identified using special stain as Grocott’s methenamine silver stain or Periodic Acid–Shiff reaction. Furthermore, to investigate specific aspects of CAPA, the questionnaire asked how many cases of invasive pulmonary aspergillosis were found and the clinical-laboratory characteristics of these patients. The questionnaire was sent in April 2021 and all responses were received after about three months. All data were subsequently collected in an Excel file. Statistical comparisons were performed with paired and unpaired chi-square tests when indicated. The data presented below include a description of the general results obtained from the survey and a brief description of findings obtained in Padova specifically focusing on previously described [3] quantitative parameters of the COVID-19-related lung lesions. Based on the presence and severity of some histological parameters, evaluated in all slides of each case scored on a ranking scale from 0 to 3 (0: absent, 1: present, focal, in <25% of the section, 2: present, ranging from 25–50%, 3: present, diffuse, in >50%), patients were categorized as pertaining to a prevalent histological phenotype. A pathological alveolar injury (AI) phenotype was found to be prevalent when the median scores of hyaline membranes, organizing pneumonia, pneumocyte type 2 hyperplasia, and squamous metaplasia were at least two times more frequent than the median scores of vascular lesions (microthrombi, large thrombi, vasculitis, and capillary inflammation). We considered the vascular injury (VI) phenotype to be prevalent when median scores of vascular lesions were two times more frequent than those of AI. A mixed phenotype was defined as having occurred when the frequency of lesions of both AI and VI were approximately the same (nearly equal median scores of the two different type of lesions).

## 3. Results

All centers responded to the survey in a period between two weeks and three months. All responses are reported in Supplementary Material (S1). Comparative data came from a total of 313 autopsies. Full autopsies were performed in five centers, limited autopsies (although always inclusive of the complete examination of both lungs) in the remaining centers. The lungs were extensively studied from a histological point of view: in seven centers more than ten lung parenchyma samples were analyzed (between four and ten in two centers), and the large airways were sampled from almost all the participants (8/9). Table 1 summarizes some demographic and clinical-pathological data, which are extensively listed in Supplementary Material (S1). Figure 1a shows the European country countries involved in the survey. Figure 1b synoptically summarizes the frequencies of the major pathological lung lesions during the two waves.

**Figure 2:**
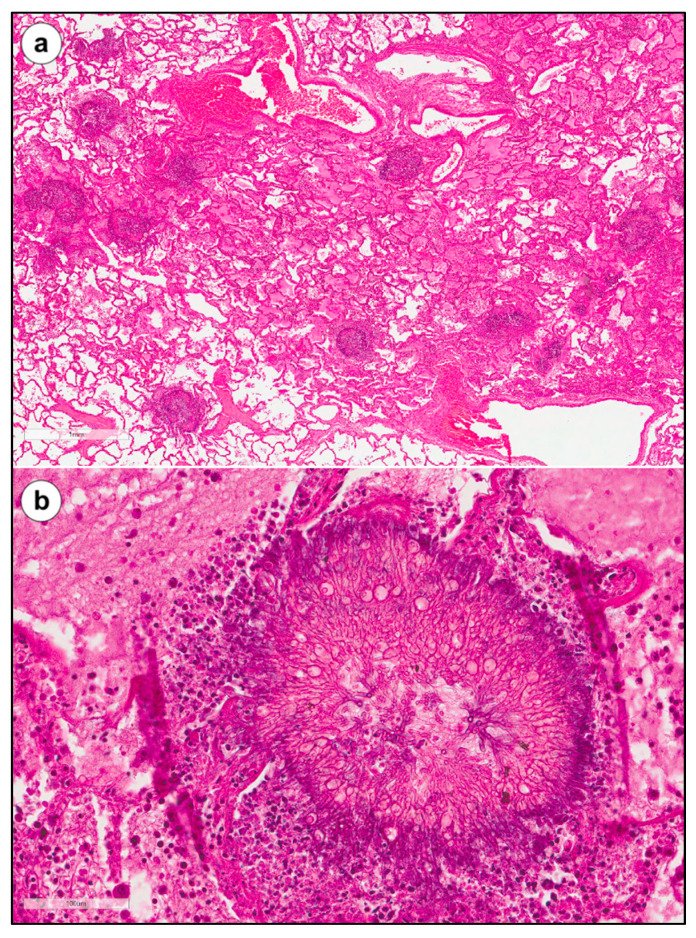
An index case of CAPA (60-year-old man) from the Padova cohort. The lung parenchyma showed oedema and several nodular aggregates that, at higher magnification, were composed of hyphae morphologically compatible with *Aspergillus* spp. ((**a**,**b**) hematoxylin and eosin, original magnification ×20 and ×200, respectively).

For most participants, DAD with vascular damage, airway inflammation, pleural involvement, emphysematous changes, and concomitant pulmonary lesions occurred with essentially equal frequency during the two waves. A higher frequency of pulmonary superinfections, both bacterial and especially fungal, was observed in the second wave compared to the first. Twenty-one cases of CAPA were reported in the second wave (21/160, 13%) and four cases in the first (4/153, 3%) (*p*-value: 0.002). The majority of CAPA patients were mechanically ventilated (18/22, 82%) and were on steroid therapy (19/22, 86%). Four patients were neutropenic (4/22, 18%) and 11 lymphopenic (11/22, 50%). Figure 2 shows the histological pictures of a CAPA index case. Interestingly, in the Padova cohort (52 autopsies)—where a quantitative analysis of different morphological lesions was performed to categorize different pathological phenotypes [3]—the AI phenotype was found to be more significantly frequent than the VI phenotype (6 VI (25%), 11 AI (46%) and seven mixed (29%)), thus becoming a completely opposite scenario compared to that of the first wave (12 VI (43%), 4 AI (14%) and 12 mixed (43%)) (*p*-value: 0.04).

## 4. Discussion

This study showed that the frequency of each lung lesion throughout the first two COVID-19 pandemic waves were somewhat heterogeneous among the participating European countries. These findings are not unexpected considering that standardized guidelines are still today found lacking when it comes to the diagnosis, management, and treatment of symptomatic patients and, especially, of hospitalized patients. Symptomatic patients, especially those who are critically ill, often showed heterogeneous and complex clinical features. Specific diagnosis and appropriate patient management are important factors that may significantly affect pathological phenotypes. Indeed, some reports have clearly found that the association of several pathological features had been significantly influenced by patient management: the occurrence of DAD was reported more frequently in patients with longer intensive care unit/hospital stays or invasive mechanical ventilation [3,10,11]. All countries involved in our survey reported a higher frequency of autoptic cases in younger ages during the second wave. Even though several studies of seroprevalence reported a younger phenotype of patients in the second wave, data of COVID-19 deaths are difficult to extrapolate as these reports may contain inaccurate accounts of the true cause of death in many countries. It is plausible that mortality in the oldest patients was lower in the second wave, however, as in this period there was preferential protection of older and high-risk people, one cannot exclude the possibility that there was an upstream selection of autopsy requests by the clinicians with a progressively lower interest in the oldest patients. The most interesting finding of the present study is that superimposed infections, especially fungal aspergillus infection, were more frequently detected. Several case reports of autoptic CAPA have emphasized this complication as being an important additional factor contributing to patient mortality [9]. Some series report a high incidence of superinfections in COVID-19 patients admitted to intensive care units: up to 40% for bacterial infections and about 20% for CAPA [8,9,10]. The higher frequency of superinfections in the second wave could be attributed to a wider use of corticosteroid therapies following the RECOVERY trial [5]. While, on the one hand, immunosuppression could have mitigated the excessive inflammatory response (i.e., “cytokine storm”), on the other hand it could have made some of these patients more susceptible to superinfections. However, possible environmental factors that could have influenced the incidence of superinfections should be considered, as the comparison between the two waves concerned different periods of the year. Since the seasonal distribution of various pathogens, in particular *Aspergillus* spp., is highly variable [12], observations over several periods are necessary to confirm our findings. In the Padova cohort, a prevalence of lesions typical of DAD (AI) was more frequently detected in the second wave. This is supported by previous clinical-pathological studies that have reported DAD as the final expression of several factors besides SARS-CoV-2 infection, such as longer disease duration and especially the occurrence of superinfections [3]. Our study has several limitations. Not all European countries were included, and the participating countries contributed with a notably different number of autopsies. However, inclusion criteria favored those centers with expertise and experience in pathology of the lungs and especially those that had an adequate number of autoptic specimens. Since our study was based just on a survey, it was impossible to investigate some clinical-pathological aspects in greater detail. Furthermore, a detailed collection of clinical, laboratory and radiological data with the inclusion of more centers is underway to obtain more precise data from clinical-pathological correlations. These data will be processed with computational analyses of extreme value to identify the potential theragnostic biomarker of the disease and will be the subject of a future article. Nonetheless, we believe that the preliminary information obtained from the survey may provide important insights into the evolution of pathological lesions through COVID-19 pandemic waves.

## 5. Conclusions

In summary, the frequency of each lung lesion was quite heterogeneous among the participants. However, a higher frequency of pulmonary superinfections, both bacterial and especially fungal, was observed in the second wave compared to the first. A deep knowledge of the pathological lesions at the basis of this complex and severe disease, which changes over time due to various factors, is crucial for correct patient management and treatment. The evidence of an increased vascular damage during the first wave made the clinicians more aware of the need to implement thromboprophylactic treatment. During the second wave, the detection of a higher frequency of CAPA led the scientific community of experts in the field to pay more attention to sensitive diagnoses of this complication. Obtaining precise diagnoses is the primary purpose of the autopsy, which remains an important medical procedure: aiding the living by understanding death (*Hic est locus ubi mors gaudet succurrere vitae*).

## Figures and Tables

**Table 1:** Study population.

	FIRST WAVE (March–August 2020)	SECOND WAVE (September 2020–February 2021)
Number of autopsies	153	160
Age range	<70: 33% 71–80: 33% >80: 33%	<70: 33% 71–80: 67% >80: 0%
Gender	Males: 85 (56%) Females: 68 (44%)	Males: 91 (57%) Females: 69 (43%)
Number of comorbidities	1: 12% 2–3: 44% >3: 44%	1: 12% 2–3: 76% >3: 12%
Number of CAPA cases	4	21

Abbreviations. CAPA: COVID-19 associated pulmonary aspergillosis

## Data Availability

The data presented in this study are available in Supplementary Material.

## Supplementary Materials

The following supporting information can be downloaded at: https://www.mdpi.com/2075-4418/12/1/95/s1, Excel S1: Clinical-pathological data from the survey.

## References

[1] Fan G., Yang Z., Lin Q., Zhao S., Yang L., He D. (2021). Decreased Case Fatality Rate of COVID-19 in the Second Wave: A study in 53 countries or regions. Transbound. Emerg. Dis..

[2] Grasselli G., Tonetti T., Protti A., Langer T., Girardis M., Bellani G., Laffey J., Carrafiello G., Carsana L., Rizzuto C. (2020). Pathophysiology of COVID-19-associated acute respiratory distress syndrome: A multicentre prospective observational study. Lancet Respir. Med..

[3] Calabrese F., Pezzuto F., Fortarezza F., Boscolo A., Lunardi F., Giraudo C., Cattelan A., Del Vecchio C., Lorenzoni G., Vedovelli L. (2021). Machine learning-based analysis of alveolar and vascular injury in SARS-CoV-2 acute respiratory failure. J. Pathol..

[4] Zarrilli G., Angerilli V., Businello G., Sbaraglia M., Traverso G., Fortarezza F., Rizzo S., De Gaspari M., Basso C., Calabrese F. (2021). The Immunopathological and Histological Landscape of COVID-19-Mediated Lung Injury. Int. J. Mol. Sci..

[5] The RECOVERY Collaborative Group (2021). Dexamethasone in Hospitalized Patients with Covid-19. N. Engl. J. Med..

[6] Saito S., Asai Y., Matsunaga N., Hayakawa K., Terada M., Ohtsu H., Tsuzuki S., Ohmagari N. (2021). First and second COVID-19 waves in Japan: A comparison of disease severity and characteristics. J. Infect..

[7] Bongiovanni M., Arienti R., Bini F., Bodini B.D., Corbetta E., Gianturco L. (2021). Differences between the waves in Northern Italy: How the characteristics and the outcome of COVID-19 infected patients admitted to the emergency room have changed. J. Infect..

[8] Koehler P., Bassetti M., Chakrabarti A., Chen S.C.A., Colombo A.L., Hoenigl M., Klimko N., Lass-Flörl C., Oladele R.O., Vinh D.C. (2021). Defining and managing COVID-19-associated pulmonary aspergillosis: The 2020 ECMM/ISHAM consensus criteria for research and clinical guidance. Lancet Infect. Dis..

[9] Fortarezza F., Boscolo A., Pezzuto F., Lunardi F., Acosta M.J., Giraudo C., Del Vecchio C., Sella N., Tiberio I., Godi I. (2021). Proven COVID-19-associated pulmonary aspergillosis in patients with severe respiratory failure. Mycoses.

[10] Buehler P.K., Zinkernagel A.S., Hofmaenner D.A., Garcia P.D.W., Acevedo C.T., Gómez-Mejia A., Shambat S.M., Andreoni F., Maibach M.A., Bartussek J. (2021). Bacterial pulmonary superinfections are associated with longer duration of ventilation in critically ill COVID-19 patients. Cell Rep. Med..

[11] Borczuk A.C., Salvatore S.P., Seshan S.V., Patel S.S., Bussel J.B., Mostyka M., Elsoukkary S., He B., DEL Vecchio C., Fortarezza F. (2020). COVID-19 pulmonary pathology: A multi-institutional autopsy cohort from Italy and New York City. Mod. Pathol..

[12] de Ana S.G., Torres-Rodríguez J.M., Ramírez E.A., García S.M., Belmonte-Soler J. (2006). Seasonal distribution of Alternaria, Aspergillus, Cladosporium and Penicillium species isolated in homes of fungal allergic patients. J. Investig. Allergol. Clin. Immunol..

